# Oral health-related quality of life, adaptation/discomfort during open bite treatment with spurs: complementary analysis from a randomized clinical trial

**DOI:** 10.1038/s41598-024-56363-0

**Published:** 2024-03-08

**Authors:** Aron Aliaga-Del Castillo, Guido Artemio Marañón-Vásquez, Guilherme Janson, Lorena Vilanova, Felicia Miranda, Camila Massaro, Silvio Augusto Bellini-Pereira, Luis Ernesto Arriola-Guillén, Marilia Yatabe, Antonio Carlos Ruellas, Lucia Cevidanes, Daniela Garib

**Affiliations:** 1 Department of Orthodontics and Pediatric Dentistry, School of Dentistry, University of Michigan, Ann Arbor, MI 48109 USA; 2 Department of Orthodontics, School of Dentistry of Ribeirão Preto, University of São Paulo, Ribeirão Preto, SP 14040 Brazil; 3 Department of Orthodontics, Bauru Dental School, University of São Paulo, Bauru, SP 17012901 Brazil; 4 Division of Orthodontics and Division of Oral and Maxillofacial Radiology, School of Dentistry, Universidad Científica del Sur, 15067 Lima, Peru; 5 Department of Orthodontics, School of Dentistry, Federal University of Rio de Janeiro, Rio de Janeiro, 21941901 Brazil; 6 Department of Orthodontics, Hospital for Rehabilitation of Craniofacial Anomalies, University of São Paulo, Bauru, SP 17012900 Brazil

**Keywords:** Orthodontics, Paediatric dentistry, Quality of life

## Abstract

This single-center trial aimed to longitudinally compare the oral health-related quality of life (OHRQOL), adaptation and discomfort during anterior open bite (AOB) treatment with lingual spurs and build-ups (SBU) versus spurs only (S) approaches. Children (7–11 years) with AOB were randomly allocated into two treatment groups (SBU or S). The Child Perception Questionnaire (CPQ_8–10_) was applied 1 and 12 months after installation of the appliances. Questionnaires evaluating functional adaptation and discomfort during the first month of treatment were also applied. A visual analog scale (VAS) was used in these questionnaires. Generalized mixed models were used for analyzing OHRQOL and discomfort data. Generalized linear models were used to assess adaptation outcomes (α = 0.05). The SBU group included 24 patients (7 males and 17 females; mean age 8.2 years) and the S group included 25 patients (11 males and 14 females; mean age 8.3 years). Regardless of the treatment type, overall OHRQOL scores at 12 months were 0.69 times those recorded at 1 month after the appliances installation (i.e., ~ 31% reduction; exp (β) = 0.69; 95% CI: 0.55, 0.88). A significant interaction between treatment and time was detected for the ‘functional limitations’ domain. For this domain, a significant improvement from the first to the twelfth month was observed in the S group (*P* < 0.001). Patients in both treatment groups showed similar and easy adaptation to the appliances. Independent of the type of treatment, tongue-related discomfort decreased over time. One week and one month after the appliance’s delivery, the discomfort scores were 0.19 (i.e., ~ 81% reduction; exp (β) = 0.19; 95% CI: 0.13, 0.28; *P* < 0.001) and 0.02 (i.e., ~ 98% reduction; exp (β) = 0.02; 95% CI: 0.01, 0.07; *P* < 0.001) times, respectively, those issued immediately after the installation of the appliances. Regardless of treatment type; overall OHRQOL improved from the first to the twelfth month of AOB treatment. The functional limitations score decreased in the S group. Children showed easy adaptation, and their discomfort decreased 1 week after the installation of the appliances.

**Trial registration:** Clinicaltrials.gov; NCT03702881, date of registration: October 11, 2018.

## Introduction

Oral health-related quality of life (OHRQOL) has important implications for orthodontics in the clinical and research fields. OHRQOL subjectively assesses how the oral conditions could affect the patient’s biopsychosocial aspects including symptoms, physical functioning, emotional and social well-being^1^. Malocclusions are directly associated with facial esthetics, function and how people are perceived by others and themselves, having a negative impact on OHRQOL^2–6^. Although some systematic reviews have shown that orthodontic treatment improves the OHRQOL^7,8^. studies with high methodological quality are still recommended on this topic.

Anterior open bite (AOB) malocclusion is characterized by the lack of contact between the incisal edges of maxillary and mandibular incisors^9^. This malocclusion impairs esthetics and causes functional problems that could expose the patients to psychosocial issues^2^. Previous studies demonstrated that AOB has a negative impact on OHRQOL^10,11^. Considering the dynamism of quality of life^12^, it is important to understand how AOB correction is perceived by the patients and how it affects their OHRQOL during treatment^7^. Currently, evidence is limited to one randomized clinical trial (RCT) that evaluated the effect of AOB correction on the OHRQOL of children^11^. It was demonstrated that treatment with palatal crib had a positive impact on the OHRQOL and that no treatment lead to a negative impact.

AOB is usually associated with increased vertical dimension. Some therapies aim to correct the habits and provide vertical control during treatment^13–17^. Spurs have been reported as a practical alternative to control the habits in young patients^18–21^ and posterior build-ups showed efficient vertical control of posterior teeth in adults^22^. The association of spurs and posterior build-ups in children is expected to correct the habit and control the vertical dimension. This combined therapy has been evaluated focusing on the dentoskeletal effects^23^ but not on patients’ perception. Spurs are usually sharpened before being installed^13,14,19,24^. Although, sharpened spurs seem aggressive, some studies have reported that patients showed overall an adequate adaptation during treatment^19,25,26^. A recent systematic review based on non-randomized studies showed very low evidence that lingual spurs have an initial transitory negative impact during interceptive treatment^27^. Thus, further RCTs are needed to evaluate patient-centered outcomes^28^, especially during AOB treatment with protocols including spurs.

### Specific objectives or hypotheses

The main objective was to longitudinally compare the OHRQOL, adaptation and discomfort during AOB treatment with bonded spurs and build-ups (SBU) versus bonded spurs only (S). The null hypothesis was that both treatment protocols demonstrate similar responses during treatment. In addition, the perception of patients regarding adaptation and discomfort were compared.

## Methods

### Trial design and any changes after trial commencement

This study was planned as a complementary outcome analysis of a previous RCT^23^, and followed the Consolidated Standards of Reporting Trials (CONSORT)^29^.

This study was approved by the Institutional Ethics in Research Committee at Bauru Dental School, University of São Paulo, Brazil (protocol no. 68551617.8.0000.5417) and was registered at Clinicaltrials.gov (NCT03702881). The authors confirm that all research was performed in accordance with relevant guidelines and regulations. Informed consent was obtained from all participants and their legal guardians. The research has been performed in accordance with the Declaration of Helsinki. Informed consent was obtained from patients and legal guardians for both study participation and publication of identifying information/images in an online open-access publication (when applicable).

### Participants, eligibility criteria, and settings

Fifty patients were recruited at Bauru Dental School, University of São Paulo, Bauru, Brazil from June 2017 to April 2018. Patients aged 7 to 11 years with AOB greater than 1 mm (clinically evaluated as the vertical distance between the incisal edges of the maxillary and mandibular central incisors)^19,30^, erupted permanent maxillary and mandibular molars and central incisors, absence or mild incisor crowding (up to 3 mm) and no need of maxillary expansion were included. All patients showed history of deleterious habits and had at least 1 deleterious habit at pretreatment. When the younger patients were evaluated for eligibility, the vertical relationship between lateral and central incisors was assessed to distinguish incomplete eruption from open bite^30^. If lateral incisors were closer to the occlusal plane than the central incisors, the condition was classified as open bite, as recommended in previous studies^14,19,30^. Exclusion criteria consisted of previous orthodontic treatment, presence of craniofacial anomalies, significant sagittal discrepancies^31^, tooth agenesis, loss of permanent teeth, moderate or severe crowding (greater than 3 mm), maxillary constriction or posterior crossbite (clinically evaluated).

### Interventions

**Figure 1:**
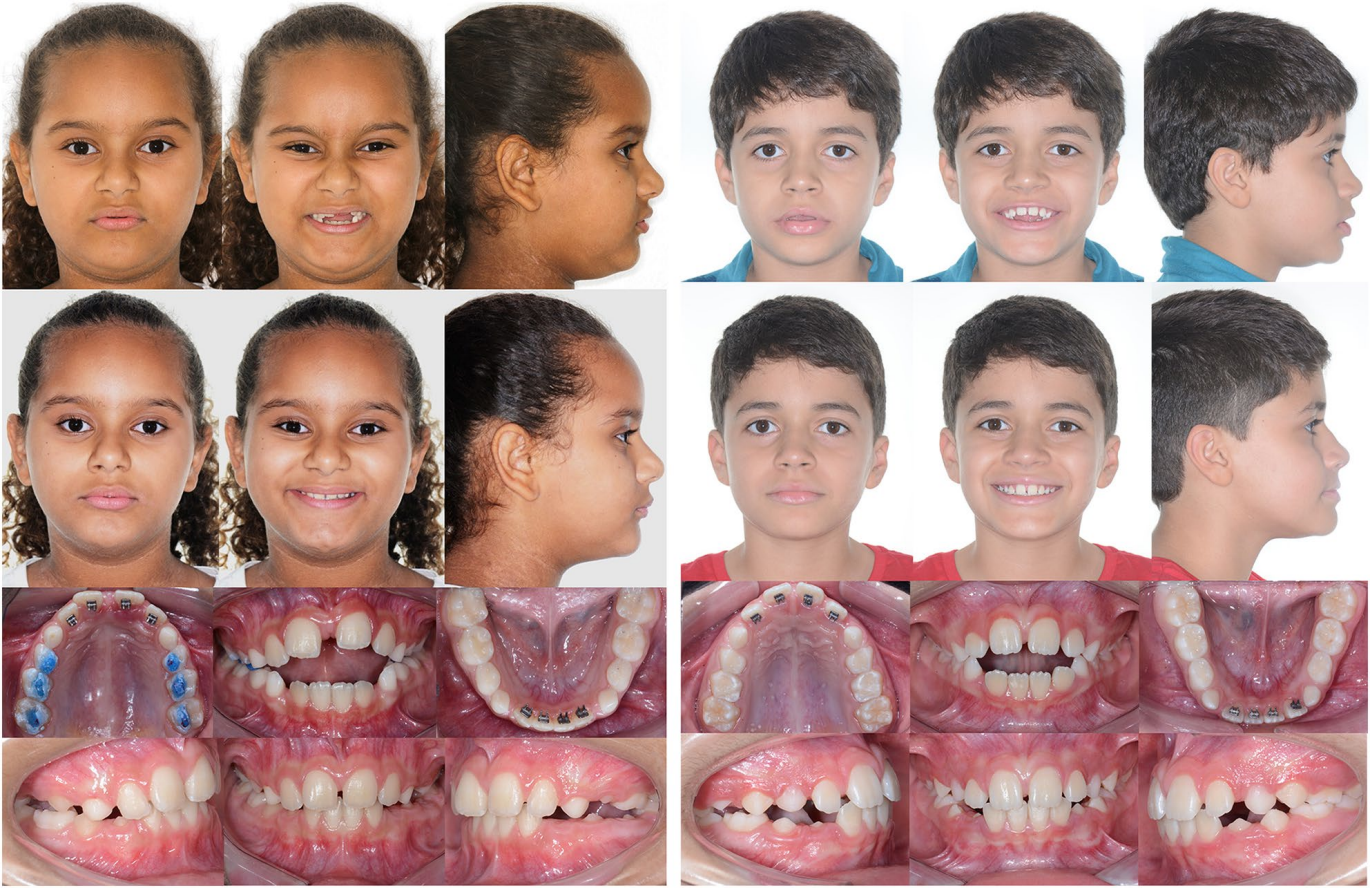
Treatment protocols. Bonded spurs associated with posterior build-ups (left) and bonded spurs only (right). Extra-oral photographs before treatment, Intra-oral photographs immediately after the installation of the appliances and after 12 months of treatment.

Two groups (SBU and S) were treated. Spurs (Morelli Ortodontia, Sorocaba, São Paulo, Brazil) were bonded at the cervical region of the lingual surfaces of maxillary and mandibular incisors of patients in both, SBU and S groups. Build-ups of 2–3 mm resin blocks (Ortho Bite; FGM Dental Products, Joinville, Santa Catarina, Brazil) were bonded on the functional cusps of the maxillary posterior teeth only in the SBU group, as described in a previous study^22^. Bite-raising was performed using resin blocks on the maxillary first permanent molars followed by resin blocks bonded on the other posterior teeth to maintain occlusal forces balance^22^ (Fig. 1). After 12 months of treatment, build-ups were removed from the SBU group and spurs were maintained in both groups as active retention. All patients and legal guardians received instructions on how to proceed in cases of spurs and build-ups debonding and/or aspiration. They should have spat them out and came to the clinic for bonding them again as soon as possible. AOB was considered corrected (closed) if the overbite was equal or greater than zero mm (end-to-end vertical incisor relationship)^15,23^. The inclusion of an untreated control group was not viable because of ethical reasons^16^.

### Outcomes

The outcomes of this complementary study included OHRQOL, adaptation and discomfort perception during treatment.

OHRQOL was evaluated using the Child Perceptions Questionnaire for 8–10 years old (CPQ_8–10_) which assesses the quality of life in 4 domains: oral symptoms, functional limitations, emotional well-being and social well-being. Questions were answered by choosing one of the 5 options related to the frequency in which conditions or situations happen in the children’s life: 0, never; 1, once or twice; 2, sometimes; 3, many times; and 4, every day or almost every day. The oral symptoms, functional limitations and emotional well-being domains contain 5 questions and the scores range from 0 to 20 in each domain. The social well-being domain contains 10 questions and the scores range from 0 to 40. The overall score ranges from 0 to 100. Zero represents no impact on OHRQOL and 100, maximum impact on children’s OHRQOL. The questionnaire also contains two questions on children’s overall perception of their oral health and general well-being with a 4-answer option each. The scores range from 0 (no impact of the oral condition on OHRQOL) to 3 (maximum impact of the oral condition on the OHRQOL). The CPQ_8–10_ Brazilian Portuguese validated version^32^ was applied after 1 month and after 12 months of treatment (just before the removal of posterior build-ups in the SBU group).

Questionnaires were also applied to evaluate adaptation to speaking, chewing, swallowing and appearance during the first month of treatment. Moreover, the level of discomfort perceived on the tongue and posterior teeth was evaluated immediately after (T0), first day (T1), 1 week (T2), and 1 month (T3) after the installation of the appliance (Supplementary Table). Questions were answered using a visual analog scale (VAS) ranging from 0 to 10 (10-cm line). For discomfort perception questions, 0 represented the minor level of discomfort and 10 the maximum level of discomfort. For adaptation questions, 0 represented the easiest adaptation and 10 the worst adaptation with the appliances. The questionnaire was applied 1-month after appliance bonding. The applied questionnaire was based on previous studies^19,25,26,33^.

The questionnaires were self-administrated. Before their application, a detailed explanation about the scoring system of each questionnaire was given to all patients and parents or legal guardians. In addition, patients were oriented to ask to their parents and to the researcher if there were not able to understand part or full questions. In these cases, a detailed explanation was provided to the patients.

### Sample size calculation

Sample size calculation was performed considering the primary outcome of this trial that was reported in a previous study^23^. A minimum of 21 participants in each group was required after sample size calculation considering the following parameters: significance level of 5%, test power of 80%, difference between groups of 1.5 mm in the overbite change (primary outcome) with a standard deviation of 1.69 mm^14^.

### Interim analyses and stopping guidelines

Not applicable.

### Randomization (random number generation, allocation concealment, implementation)

The randomization was generated using random block sizes (http://www.randomization.com)^34^. Sequentially numbered, opaque and sealed envelopes containing cards with the assigned treatment inserted into foil were used for allocation concealment. The envelopes were prepared before trial commencement. The name of the patients was written on the envelope before they were torn open. Random number generation, allocation concealment and implementation were performed by different persons^35^.

### Blinding

Blinding was not feasible because patients and the operator knew the type of appliance installed. The questionnaires were unidentified before creating the database and the statistical analysis was performed by a different person that was not involved with the randomization or treatment^36^.

### Statistical analyses

Descriptive statistics were used to present the characteristics of the groups. Generalized mixed models (GMM) were fitted to evaluate longitudinal data on OHRQOL and discomfort. The treatment type, the evaluation time and the interaction between both factors were considered as fixed effects, while the participants were considered as a random intercept. Generalized linear models (GLM) were implemented to assess data on adaptation outcomes. The treatment type was considered as fixed effect in these models. GMM and GLM were adjusted for sex, age, initial overbite, and amount of overbite correction. The Bonferroni post hoc test was applied for multiple comparisons if the GMM indicated significance for the treatment × time interaction. All statistical analyses were performed using Jamovi 2.3 software (https://www.jamovi.org). Statistical significance was set at *P* < 0.05.

## Results

### Participant flow

**Figure 2:**
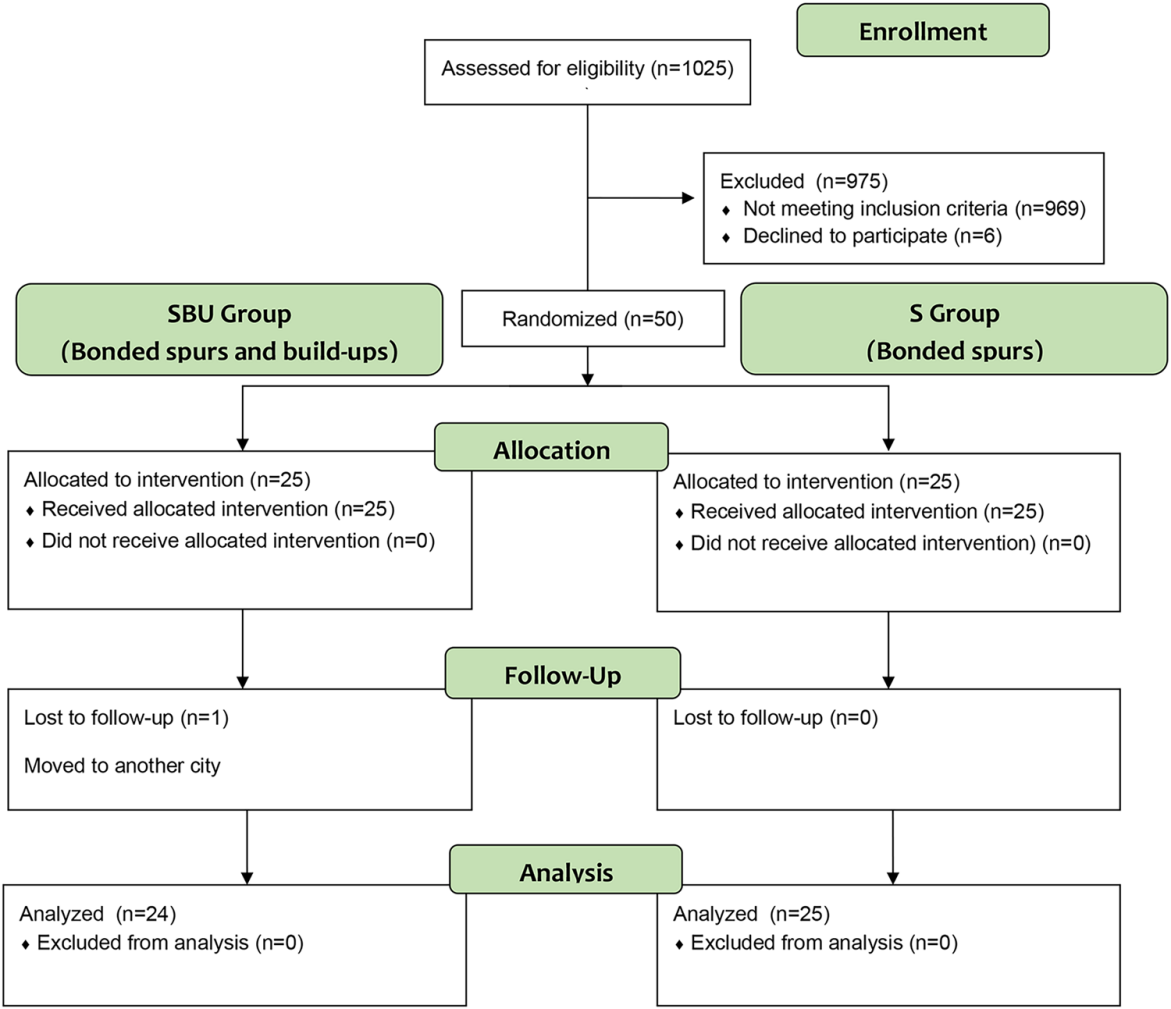
Consolidated Standards of Reporting Trials flow diagram.

Patient recruitment was performed from June of 2017 to April of 2018. After the evaluation of 1025 children, 50 patients were randomized in a 1:1 ratio (Fig. 2). The other children did not satisfy the inclusion criteria (969 patients) or decided to not participate in the study (6 patients). The SBU group had one patient lost to follow-up. Twenty-four patients in the SBU group and 25 patients in the S group were analyzed respecting their original assigned groups and using a per-protocol basis.

### Baseline data

Data regarding sex, age, overbite at pretreatment and overbite correction are described in Table 1.

**Table 1:** Characteristics of the groups: sex, age, initial overbite, overbite correction.

Variable	S group (n = 25)	SBU group (n = 24)
Sex—n (%)		
Male	11 (44.0)	7 (29.2)
Female	14 (56.0)	17 (70.8)
Age (y)—mean (SD)	8.3 (1.0)	8.2 (1.1)
Initial overbite (mm)—mean (SD)	− 4.4 (1.7)	− 4.5 (1.5)
Overbite correction (mm)—mean (SD)	4.8 (1.4)	4.8 (1.8)

*S* bonded spurs only, *SBU* bonded spurs associated with posterior build-ups, *SD* standard deviation.

### Number analyzed for each outcome, estimation, and precision

After the evaluation period, the success rate for open bite correction were 66.7% and 72% for the SBU (16/24) and S (18/25) groups, respectively. Descriptive data on OHRQOL, adaptation and discomfort is presented in Table 2.

**Table 2:** Descriptive data on Oral Health-Related Quality of Life (OHRQOL), adaptation and discomfort outcomes.

Variable	S group (n = 25)	SBU group (n = 24)
OHRQOL outcomes (CPQ_8–10_ scores)		
One month after treatment—median (IQR)		
Oral health indicator	1.0 (2.0)	1.0 (1.3)
General well-being indicator	0.0 (1.0)	0.0 (1.0)
OHRQOL domains		
Oral symptoms	4.0 (3.0)	4.0 (1.8)
Functional limitations	3.0 (3.0)	2.0 (3.3)
Emotional well-being	0.0 (2.0)	0.0 (1.3)
Social well-being	1.0 (2.0)	1.0 (2.3)
Overall OHRQOL	10.0 (8.0)	7.5 (8.0)
Twelve months after treatment—median (IQR)		
Oral health indicator	1.0 (1.0)	0.5 (1.0)
General well-being indicator	0.0 (1.0)	0.0 (1.0)
OHRQOL domains		
Oral symptoms	3.0 (5.0)	3.0 (3.3)
Functional limitations	0.0 (2.0)	0.5 (2.0)
Emotional well-being	0.0 (1.0)	0.0 (1.3)
Social well-being	0.0 (1.0)	0.0 (1.3)
Overall OHRQOL	6.0 (9.0)	5.5 (5.5)
Adaptation outcomes, 1 month after treatment—median (IQR)		
Speaking	2.0 (3.0)	2.0 (3.3)
Chewing	2.0 (3.0)	3.0 (3.3)
Swallowing	1.0 (4.0)	1.5 (3.3)
Appearance	0.0 (0.0)	0.0 (1.0)
Discomfort outcomes		
Immediately after installation (T0)—median (IQR)		
Tongue-related discomfort	3.0 (4.0)	4.0 (5.3)
Posterior teeth-related discomfort	0.0 (0.0)	0.0 (1.3)
One day after installation (T1)—median (IQR)		
Tongue-related discomfort	3.0 (3.0)	3.5 (4.0)
Posterior teeth-related discomfort	0.0 (0.0)	0.0 (2.0)
One week after installation (T2)—median (IQR)		
Tongue-related discomfort	0.0 (0.0)	0.0 (1.0)
Posterior teeth-related discomfort	0.0 (0.0)	0.0 (0.0)
One month after installation (T3)—median (IQR)		
Tongue-related discomfort	0.0 (0.0)	0.0 (0.0)
Posterior teeth-related discomfort	0.0 (0.0)	0.0 (0.0

*S* bonded spurs only, *SBU* bonded spurs associated with posterior build-ups, *SD* standard deviation, *OHRQOL* Oral Health-Related Quality of life, *CPQ* Child Perception Questionnaires, *IQR* interquartile range.

The time had a significant effect on OHRQOL (*P* = 0.002; Table 3). Regardless of the treatment type, overall scores at 12 months were 0.69 times those recorded 1 month after the appliances were installed (i.e., ~ 31% reduction; exp (β) = 0.69; 95% CI: 0.55, 0.88; Table 3). When independently evaluating each of the OHRQOL components, a significant interaction between treatment and time was detected for the ‘functional limitations’ domain (*P* = 0.011; Table 3). Post hoc pairwise comparisons found a statistically significant difference between the first and twelfth month ‘functional limitations’ scores of the S group (i.e., ~ 75% reduction; exp (β) = 0.25; *P* < 0.001). For the SBU group, the improvement of the same domain from the first to twelfth month was not statistically significant (*P* = 0.083).

**Table 3:** Estimated effects of treatment, time, and interaction of both factors on the overall perception indicators, OHRQOL individual domains, and overall OHRQOL.

Variables	Oral health indicator			General well-being indicator			OHRQOL domains												Overall OHRQOL		
							Oral symptoms			Functional limitations			Emotional well-being			Social well-being					
	Exp (β)	95% CI	*P* value	Exp (β)	95% CI	*P* value	Exp (β)	95% CI	*P* value	Exp (β)	95% CI	*P* value	Exp (β)	95% CI	*P* value	Exp (β)	95% CI	*P* value	Exp (β)	95% CI	*P* value
Treatment																					
S	Reference			Reference			Reference			Reference			Reference			Reference			Reference		
SBU	0.79	0.53, 1.20	0.271	0.71	0.37, 1.38	0.315	0.94	0.69, 1.27	0.665	1.08	0.62, 1.90	0.784	0.84	0.33, 2.13	0.707	1.05	0.52, 2.13	0.887	0.97	0.65, 1.46	0.88
Time																					
1 mo	Reference			Reference			Reference			Reference			Reference			Reference			Reference		
12 mo	0.90	0.60, 1.35	0.610	1.12	0.65, 1.94	0.686	0.92	0.75, 1.12	0.414	0.39	0.28, 0.54	< 0.001*	0.67	0.25, 1.81	0.426	0.55	0.27, 1.13	0.102	0.69	0.55, 0.88	0.002*
Treatment × time																					
S vs. SBU—1 mo	Reference			Reference			Reference			Reference			Reference			Reference			Reference		
S vs. SBU—12 mo	0.57	0.25, 1.28	0.173	1.42	0.47, 4.26	0.535	1.03	0.69, 1.54	0.883	2.27	1.20, 4.28	0.011*	1.90	0.32, 11.37	0.483	2.21	0.54, 9.15	0.273	1.40	0.87, 2.25	0.168

*CI* confidence interval, *OHRQOL* oral health-related quality of life, *S* bonded spurs only, *SBU* bonded spurs and build-ups, *mo* month(s).

Generalized mixed models adjusted for sex, age, initial overbite, and amount of overbite correction.

*Statistically significant at *P* < 0.05.

Patients showed easy adaptation to speaking, chewing, swallowing, and appearance (Table 2). No effect of treatment type on adaptation was detected (Table 4). Regarding discomfort, only a time effect was evidenced, regardless of the type of treatment implemented (Table 5). Tongue-related discomfort scores decreased over time. One week and one month after the installation of the appliances, the discomfort scores were 0.19 (i.e., ~ 81% reduction; exp (β) = 0.19; 95% CI: 0.13, 0.28; *P* < 0.001) and 0.02 (i.e., ~ 98% reduction; exp (β) = 0.02; 95% CI: 0.01, 0.07; *P* < 0.001) times, respectively, those issued immediately after installation of the appliances.

**Table 4:** Estimated effects of treatment on adaptation outcomes evaluated after 1 month of treatment.

Treatment	Speaking			Chewing			Swallowing			Appearance		
	Exp (β)	95% CI	*P* value	Exp (β)	95% CI	*P* value	Exp (β)	95% CI	*P* value	Exp (β)	95% CI	*P* value
S	Reference			Reference			Reference			Reference		
SBU	1.24	0.69, 2.22	0.482	1.03	0.63, 1.69	0.901	0.98	0.49, 1.97	0.949	1.40	0.34, 6.00	0.628

*CI* confidence interval, *S* bonded spurs only, *SBU* bonded spurs and build-ups.

Generalized linear models adjusted for sex, age, initial overbite, and amount of overbite correction.

**Table 5:** Estimated effects of treatment, time, and interaction of both factors on discomfort outcomes.

Variables	Tongue-related discomfort			Posterior teeth-related discomfort		
	Exp (β)	95% CI	*P* value	Exp (β)	95% CI	*P* value
Treatment						
S	Reference			Reference		
SBU	1.67	0.82, 3.39	0.157	^†^	0.00^†^	0.701
Time						
Immediately after	Reference			Reference		
1 day	1.01	0.81, 1.25	0.955	^†^	0.00^†^	0.774
1 week	0.19	0.13, 0.28	< 0.001*	0.80	0.00^†^	0.995
1 month	0.02	0.01, 0.07	< 0.001*	1.06	0.00^†^	0.999
Treatment × time						
S vs. SBU—immediately after	Reference			Reference		
S vs. SBU—1 day	0.99	0.64, 1.51	0.952	0.00	0.00^†^	0.767
S vs. SBU—1 week	1.77	0.83, 3.77	0.138	0.11	0.00^†^	0.974
S vs. SBU—1 month	3.41	0.37, 31.13	0.277	0.01	0.00^†^	0.955

*CI* confidence interval, *S* bonded spurs only, *SBU* bonded spurs and build-ups.

Generalized mixed models adjusted for sex, age, initial overbite, and amount of overbite correction.

*Statistically significant at *P* < 0.05.

^†^Number out of range.

### Harms

Harms were related to spurs and build-ups debonding and/or aspiration. All patients and legal guardians received instructions on how to proceed in these cases. Adaptation to speaking, chewing, and swallowing with the appliances and the temporary discomfort associated with treatment were the focus of the present study and were not considered as harms.

## Discussion

### Main findings in the context of the existing evidence and interpretation

Although the negative impact that malocclusion has on children’s OHRQOL^2–6^, limited high-quality evidence has been reported related to patients’ perception during orthodontic treatment. Most of the outcomes evaluated in orthodontic research focused on morphologic changes and do not address patients’ perspectives^28^. Especially, there is only one RCT that explored the longitudinal impact of AOB treatment with a palatal crib in OHRQOL during treatment compared with no treatment, reinforcing the need for more prospective clinical trials^11^.

Besides the palatal crib, there are various alternatives to treat children with AOB; among them, bonded spurs have been reported as an effective and practical treatment option^14,19,21^. However, no strong evidence regarding patients’ perceptions within this protocol has been reported^27^. In this regard, the present study brings important information comparing the impact of alternative approaches including lingual spurs during AOB correction in the mixed dentition. The results of this RCT provides high quality evidence on this topic.

In this study, two treatment protocols were used to correct the AOB. Both protocols used spurs and the SBU additionally used posterior build-ups to control the vertical development of posterior teeth. The effects of these protocols in the craniofacial and dentoalveolar structures have been previously reported^23,37^. AOB was corrected mainly by dentoalveolar effects, lingual inclination and extrusion of incisors and anterior dentoalveolar vertical development. Similar effects were reported in previous studies^14,18–20^. This study focused on the OHRQOL evaluated 1 and 12 months after the installation of the appliances. Adaptation and discomfort perceived during the first month of treatment were also evaluated.

The evaluation time had a statistically significant effect on the overall OHRQOL (Table 3). Among all domains, the improvement on functional limitation domain was apparently the most important contributor to the observed overall effect. Functional limitations include questions that associate teeth with difficulty for eating, chewing, speaking and sleeping^32^. It could be speculated that 1 month after the start of treatment, patients still experience functional problems associated with the intraoral appliances. Previous studies reported that during orthodontic treatment, the OHRQOL can worse slightly because of the appliance discomfort^7,38^. This temporary worsening due to the functional limitation because of the presence of the appliance was also reported during crossbite treatment with rapid maxillary expansion in children^39^. No significant occlusal vertical correction of AOB should be expected 1 month after the appliance installation^14,19^. Functional problems during speaking, chewing and swallowing are usually observed in AOB malocclusion due to the lack of contact between anterior teeth and anterior tongue posture. An altered functional pattern is present and is mainly caused by deleterious habits^9^. When the spurs are placed in the mouth, the imbalance caused by the altered function is broken and patients can experience greater functional problems due to limitations on anterior tongue posture during function. Therefore, functional limitations at 1 month can be associated with the presence of the spurs and AOB. This finding should be confirmed in future studies that include pretreatment assessments and an ideal untreated control group.

The improvement of functional limitations from the first to twelfth month of treatment observed in this study can be explained because of the expected patients’ adaptability to the appliances and clinically significant correction of AOB. Correction of AOB creates an adequate morphologic environment for an adequate function^19,25,26^. This was also reported to occur during other orthodontic treatments. OHRQOL values can be higher during the first weeks of treatment, but they progressively decrease with malocclusion correction^7,11,38,39^. These findings point to the dynamism of OHRQOL evaluation^12^.

The significant interaction between treatment and time detected for the functional limitation domain showed a statistically significant decrease of ‘functional limitations’ scores from the first to the twelfth month in the S group only. It could be speculated that the presence of posterior build-ups can cause greater functional limitations, partially restricting a significant improvement on this domain in the SBU group. Improvements of functional limitations with time in AOB treatment with spurs have been reported in previous studies^19,25,26^. In AOB patients, anterior tongue posture is critical. Spurs directly act on tongue posture and function. Their effect has been reported to have a neurophysiologic basis for changing tongue position and function, establishing a new neuromuscular pattern with time^13,24^. This can improve the functional limitations. In addition, one study evaluating tongue pressure during AOB with palatal crib therapy showed significant decreases in resting and swallowing tongue pressures, suggesting tongue adaptation during treatment^40^. Future RCTs with spur therapy associating these evaluations should be performed. It could be thought that after the removal of the spurs, the improvements in OHRQOL might be even greater^38^.

An additional questionnaire was used in this study to further evaluate the adaptation and discomfort during the first month of treatment^19,25,26^. Patients demonstrated easy adaptation to speaking, chewing, swallowing and appearance during the first month of treatment^41^, with similar results in both groups (Tables 2 and 4). This has been reported in previous studies and reinforces the easy adaptability that children can have during orthodontic treatment with spurs^19,25,26^. Within the easy adaptation range, higher scores were obtained for chewing and lower scores for appearance. Scores for chewing and appearance were expected to be higher in the SBU group because of the presence of the build-ups in the maxillary posterior teeth. However, no significant effect of treatment type on any adaptation outcome was detected.

Tongue-related discomfort and Posterior teeth-related discomfort were evaluated in both groups immediately after (T0), first day (T1), 1 week (T2) and 1 month (T3) after the installation of the appliance (Table 2). A time effect was evidenced only for Tongue-related discomfort variable, independent of the treatment type (Table 5). Similar discomfort on the tongue between groups were expected because both groups had lingual bonded spurs. Tongue-related discomfort scores are expected to decrease over time. Greater reduction of discomfort can be expected 1 week and even more 1 month after the installation of the appliances. Spurs caused discomfort on the tongue during the first days but this discomfort progressively decreases during the first month of treatment, as previously reported^19,25,26^. This initial and temporary discomfort was also reported for other orthodontic therapy in children^39^. A greater discomfort on the posterior teeth was expected for the SBU group because of the presence of build-ups. However, no effect of treatment type or time was evidenced for posterior teeth-related discomfort (Table 5).

Patient-centered outcomes are very important to be analyzed because they assist clinicians to understand how the treatment approaches are perceived by the patients and how they affect their OHRQOL. The instruments used in this study were in accordance with previous reports^11,19,25,26,32,33^. The questionnaires were self-administered avoiding any type of coercion during answering. A very detailed explanation about the questions and scoring system was given to all patients and parents. Patients were able to ask regarding doubts to their parents and/or the researcher at any moment. The understanding of the questions was double checked by the researcher.

Overall OHRQOL scores improves during AOB correction with spurs, associated or not with posterior build-ups, from the first month to twelfth month of treatment. Patients easily adapted to treatment with spurs during the first month of treatment. Discomfort was present during the first days and is expected to decrease after 1 week of treatment. The importance of the findings of this study is related to the evaluation of patient-centered outcomes using a high-quality study design (RCT) that usually is lacking in this specific population. This study brings new information regarding OHRQOL during AOB treatment in children and reinforces previous findings related to adaptation and discomfort with spurs. Communication between clinicians, patients and their legal guardians regarding what patients will experience during treatment is strongly recommended. Considering the patient-centered outcomes from the present study, the success rates on AOB correction, the similar skeletal and dentoalveolar effects between spurs only and spurs/build-ups^23,37^, spurs only therapy seems to be the more practical and efficient treatment alternative for AOB patients.

### Limitations

OHRQOL and discomfort are dynamic^11,12,33^. Ideally, OHRQOL should be also evaluated before treatment and at least 1 month after the removal of the appliances. This study attempted to measure the impact of the interventions using the CPQ_8–10_ from the first to the twelfth month of treatment. The OHRQOL questionnaire was only applied one and 12 months after the installation of the appliances. Spurs were maintained in both groups after 12 months, as active retention in patients that showed correction and as active treatment in patients that still needed some correction. Thus, evaluation after full appliance removal was not possible in this planned 12-month assessment. Although OHRQOL data from additional time points would be beneficial to have a complete longitudinal assessment of the effects of the treatment approaches on OHRQOL, similar results between groups including greater improvement on functional limitations and overall scores of OHRQOL would be expected^38^. A previous study evaluated OHRQOL of children with anterior open bite (AOB) before, 3 months after appliance delivery (fixed palatal crib) and 1 month after appliance removal^11^. The authors showed that correction of AOB had a positive impact on their OHRQOL, whereas the failure to treat this condition had a negative impact. Extrapolating the results of the previous study^11^, a decrease on these scores would be expected from pretreatment to 12 months after the installation of the appliances. Nonetheless, future studies should involve examining patients both before appliance delivery and after appliance removal.

### Generalizability

This single-center study included patients from a specific age range. Thus, the results of this study should not be generalized to children with other ages or treated with different therapies.

## Conclusions

- Similar improvements on overall OHRQOL, adaptation and discomfort were observed during AOB treatment with spurs and build-ups or with spurs only.
- Regardless of treatment type, overall OHRQOL improved from the first to the twelfth month of AOB treatment.
- Children showed easy adaptation to treatment protocols during the first month of treatment. Some discomfort was present during the first days of treatment but it decreased after 1 week of treatment.

### Supplementary Information

Supplementary Table 1.

## Data Availability

The data analyzed during the current study are available from the corresponding author on a reasonable request.
