# Supplementary material for: Oral health-related quality of life, adaptation/discomfort during open bite treatment with spurs: complementary analysis from a randomized clinical trial
Source: Sci Rep. 2024 Mar 8;14:5732. doi: 10.1038/s41598-024-56363-0 (PMC10923863; doi:10.1038/s41598-024-56363-0)
Supplement: Supplementary file 1 — Supplementary Table 1. [file 41598_2024_56363_MOESM1_ESM.docx]

**Supplementary Table.** Questions used to evaluate adaptation and discomfort.

| **Adaptation Questions:** |
| --- |
| **Q1.** How has it been adjusting to the appliance when **speaking**? |
| **Q2.** How has it been adjusting to the appliance when **chewing**? |
| **Q3.** How has it been adjusting to the appliance when **swallowing**? |
| **Q4.** How has it been adjusting to the appliance in terms of **appearance** (esthetically)? |
|  |
| **Discomfort Questions:** |
| **Q5.** How much discomfort did you feel on your **tongue** **immediately** **after** the delivery of the appliance? |
| **Q6.** How much discomfort did you feel on your **posterior teeth immediately after** the delivery of the appliance? |
| **Q7.** How much discomfort did you fell on your **tongue 1 day after** the delivery of the appliance? |
| **Q8.** How much discomfort did you feel on your **posterior teeth 1 day after** the delivery of the appliance? |
| **Q9.** How much discomfort did you feel on your **tongue** **1 week after** the delivery of the appliance? |
| **Q10.** How much discomfort did you feel on your **posterior teeth 1 week after** the delivery of the appliance? |
| **Q11.** How much discomfort do you feel on your **tongue 1 month after** the delivery of the appliance? |
| **Q12.** How much discomfort do you feel on your **posterior teeth 1 month after** the delivery of the appliance? |
